# Viral Hepatitis and Iron Dysregulation: Molecular Pathways and the Role of Lactoferrin

**DOI:** 10.3390/molecules25081997

**Published:** 2020-04-24

**Authors:** Romina Mancinelli, Luigi Rosa, Antimo Cutone, Maria Stefania Lepanto, Antonio Franchitto, Paolo Onori, Eugenio Gaudio, Piera Valenti

**Affiliations:** 1 Department of Anatomical, Histological, Forensic Medicine and Orthopedic Sciences, Sapienza University of Rome, 00185 Rome, Italy; antonio.franchitto@uniroma1.it (A.F.); paolo.onori@uniroma1.it (P.O.); eugenio.gaudio@uniroma1.it (E.G.); 2 Department of Public Health and Infectious Diseases, Sapienza University of Rome, 00185 Rome, Italy; luigi.rosa@uniroma1.it (L.R.); mariastefania.lepanto@uniroma1.it (M.S.L.); piera.valenti@uniroma1.it (P.V.); 3 Department of Biosciences and Territory, University of Molise, 86090 Pesche, Italy; antimo.cutone@unimol.it; 4 Eleonora Lorillard Spencer Cenci Foundation, 00185 Rome, Italy

**Keywords:** liver, viral infections, hepatitis, iron, iron homeostasis, iron proteins, lactoferrin, lactoferrin receptors, hepcidin, ferroportin

## Abstract

The liver is a frontline immune site specifically designed to check and detect potential pathogens from the bloodstream to maintain a general state of immune hyporesponsiveness. One of the main functions of the liver is the regulation of iron homeostasis. The liver detects changes in systemic iron requirements and can regulate its concentration. Pathological states lead to the dysregulation of iron homeostasis which, in turn, can promote infectious and inflammatory processes. In this context, hepatic viruses deviate hepatocytes’ iron metabolism in order to better replicate. Indeed, some viruses are able to alter the expression of iron-related proteins or exploit host receptors to enter inside host cells. Lactoferrin (Lf), a multifunctional iron-binding glycoprotein belonging to the innate immunity, is endowed with potent antiviral activity, mainly related to its ability to block viral entry into host cells by interacting with viral and/or cell surface receptors. Moreover, Lf can act as an iron scavenger by both direct iron-chelation or the modulation of the main iron-related proteins. In this review, the complex interplay between viral hepatitis, iron homeostasis, and inflammation as well as the role of Lf are outlined.

## 1. The Liver as Central Immunological Organ

The liver represents not only the largest visceral organ in our body but also an essential immunological organ, playing several crucial roles including detoxification, protein synthesis, and bile production [1]. The liver also exerts storage and recycling functions for many metabolites, vitamins, and minerals, including iron. Iron metabolism and homeostasis critically rely on the liver, being the major site for (i) the production of proteins that maintain systemic iron balance; (ii) the storage and mobilization of iron from hepatocytes to the circulation to meet metabolic requirements; (iii) iron recycling in conjunction with the spleen (splenic macrophages) [2].

Receiving both portal vein blood and arterial blood, the liver is a central component in the defense against blood-borne infections [3]. Oxygen-rich arterial blood enters the liver through the hepatic artery (HA). The greater part of blood (80%) entering the liver is supplied by the portal vein (PV), mainly coming from the intestine. The liver is exposed to non-self-proteins derived from nutrients or resident microbiota, which, combined with the constant presence of bacterial endotoxins, could normally trigger immune responses [4,5]. Injury to the liver, such as viral hepatitis, alcohol abuse or drug toxicity, and cholestatic diseases, leads to hepatic inflammation, which could induce the development of chronic liver disease and fibrosis [6]. For that reason, the hepatic capillary system is lined with different specialized cells such as the sinusoidal endothelial cells (LSECs), the largest population of macrophages or resident Kupffer cells (KCs), natural killer T (NKT) cells, and the largest reticulo-endothelial cell network [7,8]. Moreover, the liver also counts the largest population of natural killer (NK) cells. These innate lymphocytes play an important role in the protection from infection and tissue pathology [9]. In addition, Toll-like receptors (TLRs) such as TLR4, classical receptors for activating the immune system, are constitutively expressed on hepatocytes, LSECs, and hepatic stellate cells (HSCs) [10,11]. Other control cells are myeloid cells, such as monocytes, which monitor the liver vasculature [12]. In normal conditions, monocyte-derived cells can develop into liver dendritic cells (DCs) or monocyte-derived macrophages (MoMFs), but they are not included in the pool of local resident macrophages (Kupffer cells) [13].

The several populations of DCs are more classically associated with T cell activation in other tissues such as the spleen and lymph nodes [14]. They can enter into the liver as immature cells through the portal vein, then they continue to mature as they move to the central vein or as they transmigrate through the LSECs to enter in the Disse’s space [15]. Although DCs are primarily responsible for antigen presentation to adaptive immune cells, macrophages act as primary filter cells [16].

KCs can also recruit other immune cells, such as monocytes, into the liver, which are differentiated into interleukin (IL)-10 positive/IL-12 negative cells by hepatocyte growth factor, macrophage colony-stimulating factor (M-CSF) [17] or low-levels of lipopolysaccharide (LPS) stimulation, inducing the activation of Signal Transducer and Activator of Transcription3 (STAT3) and Smad and then inducing the blocking of NF-κB [18].

Plasmacytoid DCs make up a specialized DC population, which is abundant in the liver, with a fundamental role in viral defense mediated by the production of type I interferons (IFN) [19]. Altogether, the liver reticulo-endothelial system forms a greatly dynamic and complex network, constituting a primary line of defense against microorganisms coming from the gut epithelial barrier. Different mechanisms are present to trigger immune reactions in the liver to employ mechanisms for rapid activation in response to infectious diseases or tissue damage.

Depending on the type of liver disease, different triggers have been identified for activating the immune system. For example, TLR3 represents one of the main triggers in the defense against viral diseases. In the crosstalk between NK cells and KCs, TLR3 activation induces a higher IFN-γ response compared to TLR2 and TLR4, probably due to the induction of IL-12 without the presence of IL-10 coproduction [20].

## 2. Viral Infections of the Liver: Hepatitis

Hepatitis represents an inflammation of the liver commonly caused by a viral infection, but this disease could also be triggered by other factors, such as alcohol, drugs, toxins, and autoimmunity. It is a condition which can be self-limiting or can progress to fibrosis, cirrhosis, or hepatocarcinoma (HCC). Viral infections are classified as hepatitis and they include hepatitis A, B, C, D, and E. Among these, the three most common types of viruses in the United States are hepatitis A virus (HAV), hepatitis B virus (HBV), and hepatitis C virus (HCV), while the remaining two types are less common and related to hepatitis D virus (HDV) and to hepatitis E virus (HEV). The latter includes different sub genotypes: genotypes 1 and 2 (GT1 and GT2) are diffused in developing countries, while GT3 and GT4 are mainly disseminated in developed countries. In most cases, HEV determines a self-limiting hepatitis. GT3 and GT4 can induce a chronic hepatitis that can rapidly result in cirrhosis in immunocompromised patients. In these conditions, reduction of immunosuppression is the first therapeutic option and the use of anti-viral therapies, such as PEGylated-interferon and ribavirin, has been found to treat HEV infection in an effective way [21,22]. Hepatitis can induce an acute illness characterized by nausea, abdominal pain, and jaundice. HBV and HCV can additionally lead to the establishment of a chronic infection correlated to an increased risk of chronic liver disease and HCC [23,24,25]. There are vaccines available for some types of hepatitis and other kinds of medical treatments are present for the types for which a vaccine is unavailable. Hepatitis with a duration of less than six months is called acute hepatitis while, when it exceeds the six-month timeframe, it is called chronic hepatitis [26]. Hepatitis A and E are acquired by the ingestion of contaminated food or water, while hepatitis B, C, and D usually develop after a parenteral contact with infected body fluids [27].

The hepatitis A is due to an RNA virus from the Picornaviridae family. The highest viral concentration is present in the stool of infected patients. In most cases, the infection is mild, and most people make a full recovery from it and become immune from other HAV infections. The infection is more common in developing countries, where it can become severe and life threatening. International travel is the most significant risk factor identified from the cases reported in the United States. The incubation period of HAV is of approximately four weeks. Acute infections of HAV are more severe and have a higher mortality rate in adults rather than children. For that reason, safe and effective vaccines are available to prevent HAV [28].

The HBV is a DNA virus, member of the Hepadnaviridae family. Three types of viral particles are discovered in infectious serum. Two of them are smaller spherical structures with filaments of variable lengths. The spheres and filaments are composed of hepatitis B surface antigen (HBsAg) and host-derived lipids without viral nucleic acids and are therefore noninfectious. The infectious HBV virion has a spherical, double-shelled structure, consisting of a lipid envelope containing HBsAg that surrounds an inner nucleocapsid composed of hepatitis B core antigen (HBcAg) complexed with virally encoded polymerase and the viral DNA genome [29,30]. It is present in and can be transmitted through exposure to infectious blood, semen, saliva and other body fluids. For that reason, HBV can be transmitted also from infected mothers to infants at the time of birth. Hepatitis B virus infected about 364 million people around the world, about 4% of the world population [31]. Prevalence was highest in East Asia and sub-Saharan Africa, reaching 12% in the Central African Republic, compared to less than 1% in the UK. Moreover, 57% of these infections were in five countries: China, India, Nigeria, Indonesia, and the Philippines [32]. In the United States, about 2.2 million people have a chronic HBV infection, transmitted parenterally and sexually when individuals come into contact with body fluids, of infected individuals, as previously mentioned. The incubation period of an acute HBV infection is around 12 weeks, with the majority of patients experiencing mild illness and less than 1% of patients with fulminant hepatic failure. In addition, to prevent HBV, safe and effective vaccines are available [33].

The HCV is an RNA virus, and a member of the Flaviviridae family with one serotype, but at least six major genotypes and more than 80 subtypes. HCV is mainly transmitted through exposure to infected blood. Modes of transmission for this virus can be parenteral, perinatal, and sexual, but the most common is through the sharing of contaminated needles during medical procedures. The incubation period of HCV is of approximately eight weeks. Most cases of acute hepatitis C infection are asymptomatic; however, about 55% to 85% of patients develop chronic hepatitis C and liver disease, and 30% of those patients end up developing cirrhosis. At the moment, there is no vaccine for HCV due to its extensive genetic variability, which makes it challenging to develop a vaccine to prevent this type of hepatitis [34]. However, a recent study has designed a trivalent vaccine containing sE2 from genotype 1a, 1b, and 3a that has induced pangenotypic NAbs in mice, which neutralized HCVcc of all the seven genotypes more potently than the monovalent vaccine [35].

Hepatitis D is due to an RNA virus, single species in the Deltavirus genus. It contains the hepatitis D antigen, the RNA strand, and it uses HBsAg as its envelope protein. Infections occur only in those who are infected with HBV. In fact, HDV has similar modes of transmission to HBV. The concomitant infection of HDV and HBV can induce a more serious disease and worse outcome. The incubation period for the HDV virus is of approximately 13 weeks. Fortunately, hepatitis B vaccines provide protection from HDV infection as well [36].

Hepatitis E is related to an RNA virus, a single species in the Orthohepevirus genus. Similar to HAV, it is mostly transmitted through consumption of contaminated water or food. HEV is a common cause of hepatitis outbreaks in developing countries. The incubation period for the HEV is of approximately 2 to 10 weeks. Although a vaccine for HEV has been developed, it is still under debate and only licensed in China. Thus, more clinical data are needed to verify whether this vaccine or any vaccines being developed work effectively against the different genotypes [37,38].

Another possible hepatic infection is represented by the autoimmune hepatitis, but its exact etiology is unknown. Several factors like drugs, environmental agents, or viral infection from hepatitis viruses may trigger an autoimmune response, in which patients develop circulating autoantibodies and gamma globulin. Most of the patients have a family history of other autoimmune disorders or have been subjected to immunosuppressive therapy in the past [39]. To complete the list, there is also alcoholic hepatitis, with a complex pathogenetic mechanism, where different factors, such as genetic factors, metabolism of ethanol and its metabolites can induce damage of hepatocyte cell membranes, malnutrition, stimulation of cytokines accelerating cell death, steatotic changes, free radicals, and oxidative injuries [40].

Importantly, chronic liver injury and histological alteration related to hepatitis, including viral, autoimmune, and alcoholic hepatitis strongly impact systemic iron homeostasis, whose imbalance can, in turn, aggravate liver injury itself. Indeed, both experimental and clinical studies have demonstrated that iron homeostasis disorders, mainly resulting in hepatic iron overload, aggravate liver injury and are associated with higher risks of developing fibrosis, cirrhosis, and hepatocellular carcinoma [41]. It is therefore of utmost importance to take into account iron homeostasis disorders when referring to hepatitis.

## 3. Systemic and Hepatic Iron Homeostasis

Before introducing iron metabolism and homeostasis, it is important to underline that iron is an essential element for living cells and it is a component of fundamental biological processes such as DNA replication and energy production in both humans and microbial pathogens. As iron is extremely insoluble, in order to acquire it, hosts have developed different iron-binding proteins which are able to establish a battle for iron chelation with microbial metallophores, secreted compounds for iron scavenging. Indeed, host iron-binding proteins play a pivotal role in the prevention of microbial growth and infection by decreasing bacterial iron availability. However, iron can be toxic when present in excess because of its capacity to donate electrons to oxygen, resulting in the generation of reactive oxygen species (ROS), which are able to damage proteins, lipid membranes, and DNA, thus inducing tissue injury and organ failure [42]. In physiological conditions, the amount of free available iron does not exceed 10^−18^ M, a much smaller concentration than that required for microbial growth, ROS formation and, consequently, the induction of the inflammatory process [43,44]. In pathological conditions, intracellular iron overload increases host susceptibility to infection, ROS formation, and inflammation [44]. In humans, this dual role of iron has led to the evolution of tight controls to avoid its toxicity. In developed countries, an equilibrated diet provides about 15 mg of iron per day but only about 10%, corresponding to 1–2 mg, is absorbed due to its exceptionally poor bioavailability. Moreover, on a daily basis, the 20 mg of iron derived from lysed erythrocytes and the few milligrams of it derived from cells such as hepatocytes and macrophages are utilized for the de novo synthesis of heme [44]. Dietary iron absorption, occurring on the apical side of enterocytes, is carried out by the combined action of a ferrireductase, the duodenal cytochrome B, DCYTB, and a permease, the divalent metal transporter 1 (DMT1). Once in the cytoplasm, ferrous iron is sequestered and reoxidated by an iron storage protein, ferritin (Ftn). The release of iron from this protein into the cytoplasm occurs after a successive reduction from ferric to ferrous ions. Ferrous ions are, then, exported into the plasma by ferroportin (Fpn), the only known mammalian iron exporter found on the cytoplasmic membrane of enterocytes, hepatocytes, macrophages, and placental cells [45]. Of note, Fpn acts in conjunction with two ferroxidases: hephaestin (Heph), found in epithelial cells; and ceruloplasmin (Cp), in hepatocytes [46] and macrophages [47]. Both ferroxidases convert ferrous into ferric ions in order to allow their binding to serum transferrin (Tf) in the blood [47].

The interface between systemic and cellular iron homeostasis, including hepatocytes, involves the cellular iron acquisition by Tf-bound iron through Tf receptor 1 (TfR1)-mediated endocytosis. Tf is an iron binding glycoprotein, divided into two lobes (N- and C-lobe), able to chelate and release iron, thus exerting an iron delivery function [48]. In the acidic endosome, ferric iron bound to Tf is released as a ferrous ion and translocated via DMT1 into the cytoplasm, where it can either be utilized for cell metabolism or sequestered by Ftn. Only for particular iron-recycling cells, such as enterocytes, hepatocytes, and macrophages, intracellular iron can be further exported into the plasma by Fpn [49]. 

**Figure 1:**
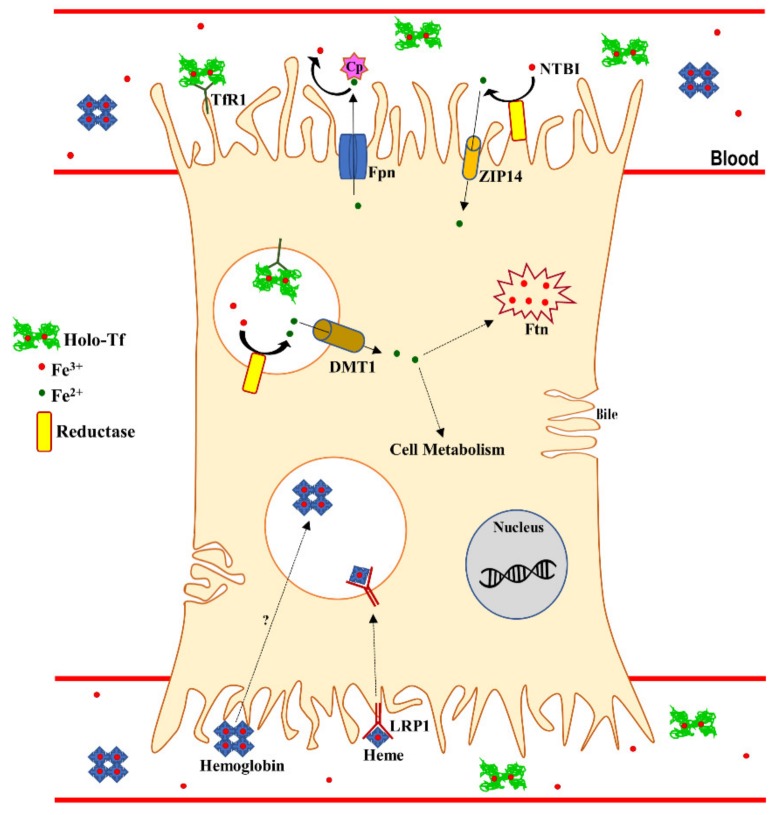
Iron uptake and excretion in hepatocytes. Tf-bound ferric iron uptake is mediated by TfR1, inducing endocytosis. Once in the acidic endosome, ferric iron is reduced by a ferric reductase and exported to the cytoplasm by DMT1. The ferrous iron is then utilized for cell metabolism or sequestered into Ftn. Acquisition of NTBI involves the reductase-mediated ZIP-4 import as ferrous iron. Moreover, heme-bound iron is imported by LRP1 or though hemoglobin internalization. Despite the numerous uptake mechanisms, iron is exported only through the Fpn/Cp system. Tf: transferrin; Holo-Tf: transferrin-bound iron; Fpn: ferroportin; Cp: ceruloplasmin; TfR1: transferrin receptor 1; NTBI: non-transferrin-bound iron; ZIP-14: ZRT/IRT-like protein-14; DMT-1: divalent metal transporter 1; Ftn: ferritin; LRP1: Low Density Lipoprotein receptor related protein. This figure has been drawn, with few modifications, according to the scheme proposed by Knutson et al. [50].

Other than by Tf, specialized cells, including macrophages and hepatocytes, can uptake iron by non-Tf-bound iron (NTBI), hemoglobin, and heme, as summarized in Figure 1.

It is well known that the liver clears excess of NTBI by its rapid uptake [51]. NTBI refers to a heterogeneous mixture of low-molecular-weight forms of iron that become detectable in plasma when Tf saturations exceed 75%. NTBI is acquired by the liver via ZRT/IRT-like protein-14 (ZIP14), a transmembrane metal-ion transporter located on the sinusoidal membrane of hepatocytes, originally identified as a zinc transporter, but subsequently also defined as an iron transporter [52].

Systemic iron homeostasis is tightly regulated by several stimuli and parameters, including iron levels and inflammation [53]. In particular, the liver plays a crucial role in iron homeostasis through the synthesis of hepcidin, a cationic peptide hormone able to bind to Fpn, thus inducing its internalization and degradation [54]. Hepcidin, synthesized by hepatocytes and released in the plasma [55], is up-regulated by iron stores [56] and pro-inflammatory cytokines, such as IL-6 [57,58,59,60].

**Figure 2:**
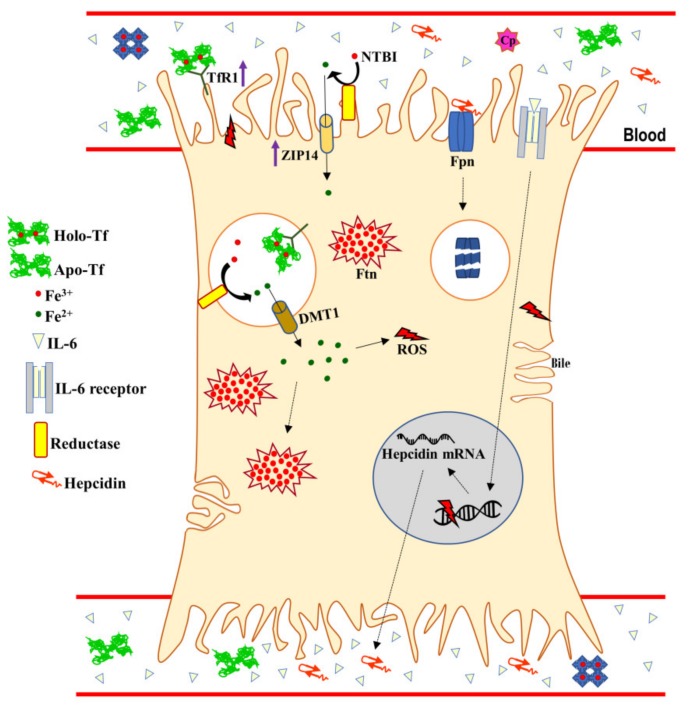
Iron uptake and excretion in inflamed hepatocytes. The liver is the major organ responsible for iron storage upon inflammatory stimuli. The iron homeostasis disorders start with the up-regulation of systemic IL-6, which stimulates the synthesis of hepcidin by the liver. Hepcidin binding to Fpn leads to the internalization and lysosomal-mediated degradation of the permease, thus blocking iron export. Accordingly, Tf-independent and -dependent iron uptake systems are up-regulated (purple arrows), guaranteeing that excess systemic iron is accumulated in Ftn. Notably, intracellular iron overload induces ROS which, in turn, damage proteins, lipid membranes, and DNA, thus inducing tissue injury and organ failure. According to intracellular iron overload, anemia of inflammation is established.IL-6: interleukin-6; Fpn: ferroportin; Tf: transferrin; Holo-Tf: saturated transferrin; Apo-Tf: unsaturated transferrin; TfR1: transferring receptor 1; Ftn: ferritin; NTBI: non-transferrin-bound iron; ZIP-14: ZRT/IRT-like protein-14; DMT-1: divalent metal transporter 1; Ftn: ferritin; ROS: reactive oxygen species; LRP1: Low Density Lipoprotein receptor related protein.

The Fpn degradation, caused by the binding to hepcidin, or its down-regulation by IL-6 provoke a significant decrease of iron export from cells into plasma and an increase of intracellular iron concentration [53,61,62]. Although the down-regulation of Fpn is shared by all the cell types specialized in absorption and iron-recycling, i.e., enterocytes, hepatocytes, and macrophages, the contribution of iron uptake systems to the establishment of intracellular iron overload is cell dependent. As matter of fact, following inflammatory stimuli, enterocytes and macrophages down-regulate TfR1 and DMT1 expressions [44], whereas hepatocytes take charge for most of the systemic iron by up-regulating both TfR1 [63] and ZIP-14 levels [64], thus clearing excess iron by increasing the internalization rates of Tf-bound iron and NTBI, respectively (Figure 2).

Altogether, these changes result in both iron overload in enterocytes, hepatocytes, and macrophages, and, at the systemic level, in iron deficiency (ID), ID anemia (IDA), and anemia of inflammation (AI) [42,65,66]. It is well known that intracellular iron overload is a pivotal parameter influencing host infections [67,68,69,70]. As a matter of fact, intracellular iron overload influences host susceptibility to bacterial and viral infections as well as together with pro-inflammatory cytokines modulates the expression of iron-related proteins such as Tf, TfR1, hepcidin, Fpn, intracellular Ftn, and lactoferrin (Lf) [44]. The iron proteins belong to the innate immune responses and are modulated not only by infection but also by inflammatory processes [53,62,68,69,71,72]. During microbial and viral infections, iron homeostasis is perturbed, leading to iron disorders [67], which are worsened by the action of pro-inflammatory cytokines [53]. In in vitro models, enterocytes infected by intracellular bacteria produce pro-inflammatory cytokines, including IL-6 [73] which, in turn, decrease Fpn synthesis [74]. Similarly, in inflamed macrophages, an iron overload has been found and it has been associated to the decrease of Fpn and TfR1 as well as to the increase of intracellular Ftn [61,62]. As already reported, intracellular iron overload is generally related to iron deficiency in the circulation, which is considered a defense mechanism to limit iron availability to pathogens in the blood [75,76]. However, considering that the bacteria can widely multiply thanks to the iron overload inside circulating macrophages, this concept of host defense should be critically reviewed. In fact, it is well known that the excess of iron can stimulate the intracellular replication of several facultative and obligate intracellular bacteria and worsen the severity of infection [72,74,77,78,79,80]. Of note, infections and related inflammations induce hepcidin up-expression, thus reducing serum iron concentration and increasing iron overload in reticuloendothelial cells [71]. When hepcidin expression increases, the iron-saturation of Tf decreases similarly to TfR1 [81] and Fpn [53]. Therefore, the host’s iron status can alter the course of infection and its resolution. Viral infections need active cell metabolism and, therefore, a significant viral replication requires a high iron availability [82]. As a general rule, intracellular iron overload, induced by up-expression of hepcidin, promotes the progression of viral infections, as demonstrated for the human immunodeficiency virus (HIV) [83]. Conversely, HCV infections represent a peculiar exception, presenting the down-regulation of hepcidin, which is then up-regulated following antiviral therapy [81]. Intracellular iron overload damages HCV particles.

Since viral infections are directly promoted by intracellular iron overload, other than the contribution of the iron export system, the involvement of the iron uptake system, Tf/TfR1, in the establishment and maintenance of intracellular iron dysregulation, cannot be neglected. TfR1, the main receptor for Tf, which is highly expressed on the plasma membrane, is a homodimeric type II membrane glycoprotein (~95 kDa) characterized by an apical, a helical, and a protease-like domain [84,85]. In particular, the N-lobe of Tf interacts with the protease-like domain, while the C-lobe interacts with the receptor’s helical domain. Interestingly, the apical domain of TfR1 is an attractive target for viral particles [86]. The canine parvovirus (CPV) and the feline panleukopenia virus (FPV) have been reported to infect host cells through TfR1 [87] as well as the mink enteritis virus [88] and the New World hemorrhagic fever arenaviruses [89,90]. HCV also interacts with TfR1 for its internalization in endosomal compartments [91]. Therefore, similar to the mechanism described for Tf, upon the binding of viral particles, TfR1 undergoes endocytosis, thus translocating viruses into intracellular compartments. Of note, the interaction between the virus and TfR1 does not interfere with iron delivery and TfR1 expression [70]. Recently, the effect of HCV infection on TfR1 recycling has been deepened [92]. In particular, HCV-infected cells showed decreased levels of α-taxilin, an essential factor for the TfR1 recycling, thus resulting in lower levels of TfR1 active protein and consequently in reduced Tf binding, internalization, recycling, and intracellular iron level [92]. Overall, while iron homeostasis disorders associated to bacterial infections have been well defined, the relationship between iron homeostasis and viral infection is still not well understood [70].

## 4. Lactoferrin and its Receptors

Among the proteins belonging to the Tf family, Lf is a glycoprotein of ca. 690 amino acid residues able to reversibly chelate two Fe (III) per molecule with high affinity (Kd~10^−20^ M). Differently from Tf, which retains iron at a pH not lower than 5.5, Lf retains ferric iron at pH values as low as 3.0. Human Lf (hLf) is a cationic molecule of the innate immunity, constitutively synthesized by exocrine glands and, following the induction, by neutrophils in infection and inflammation sites [44]. Of note, 10^6^ neutrophils release 15 µg of Lf. HLf is divided into two homologous lobes (N-lobe, residues 1–333, and C-lobe, residues 345–691) connected by a 3-turn α-helix peptide (residues 334–344) [93,94]. Each lobe, constituted by two domains (N1 and N2, C1 and C2), binds one ferric ion and one carbonate anion within a deep cleft between the domains of each lobe [94]. The Fe(III) ligands, which are highly conserved among most iron-binding proteins [95,96], are identical in both lobes: one aspartic acid, two tyrosines, and one histidine (Asp-60, Tyr-92, Tyr-192 and His-253 in the N-lobe and Asp-395, Tyr-433, Tyr-526 and His-595 in the C-lobe). Lf can adopt two main conformational states: the open metal-free (apo-Lf) and the closed metal-bound (holo-Lf). Interestingly, the iron content influences the physico-chemical properties of Lf [97]. Metal binding and release are, therefore, related to large-scale conformational changes [98]. These lobes are unequally glycosylated; in fact, the majority of glycosylation sites are present in the C-lobe. HLf contains three potential *N*-glycosylation sites, located at Asn138, Asn479, and Asn624 [99,100]. Even if Lf is highly conserved among human, bovine, mouse, porcine, and camel species, the highest sequence homology has been identified between human and bovine Lf (bLf) [101]. BLf, sharing about 70% of sequence homology with hLf, possesses five glycosylation sites at Asn−233, −281, −368, −476, and −545 [102] and shares identical biological functions with hLf [44]. For this reason, bLf is used in both in vitro and in vivo studies being available in large quantities and being generally recognized as safe (GRAS) by the Food and Drug Administration (FDA). The multiple functions of Lfs rely on their ability to chelate two ferric ions and to bind to abiotic or biotic anionic surfaces [103]. The ability to bind iron reduces the availability of this essential nutrient for microbial pathogens. As already mentioned, many bacterial and fungal infections are influenced by iron availability for growth and host colonization. Several studies have demonstrated that high iron concentrations are also positively associated with viral infections, as demonstrated by the increase of HIV load [104] and mortality rate [105,106]. Therefore, bLf inhibits HIV infection [107,108]. Other than anti-viral and anti-microbial activities [44,109,110], both hLf and bLf perform other pivotal biological activities, such as anti-inflammatory, immunomodulatory, and anti-cancer ones [111,112,113]. The anti-inflammatory activity exerted by Lf is strictly dependent on its ability to enter inside host cells through receptor-mediated endocytosis and to consequently translocate into the nucleus [114], thus modulating pro-inflammatory gene expression in the host cells [115,116]. In fact, the binding between hLf and its receptors (hLfRs) can activate different intracellular signaling pathways [117] or result in the hLf clathrin-mediated endocytosis and subsequent nuclear translocation [114], thus enabling hLf to act as a regulator or trans regulator of cellular gene expression [118]. It has been also found that hLfRs, which are differentially expressed in different tissues and cell types, are related to multifunctional activities of hLf [119]. In 1991, the first human intestinal receptor from fetal intestinal brush-border membranes was isolated [120], while, in 2001, this receptor was cloned and functionally studied [121]. This receptor is currently known as intelectin-1 (ITLN1) [122]. ITLN1 is a glycoprotein constituted of three 40 kDa subunits that are cross-linked by disulfide bonds making up a 120 kDa homo-trimer of 295 amino acids and N-linked oligosaccharides [122]. This receptor is present in the intestinal epithelium [119], in Paneth and goblet cells [123], as well as on cholangiocytes [124]. ITLN1 is a high affinity Lf receptor (Kd = 10^−6^ M) able to transduce several Lf-mediated functions, ranging from the facilitation of intestinal iron absorption in infants to the strengthening of the immune system [125,126,127,128]. Besides ITLN1, Low Density Lipoprotein (LDL) receptor related protein (LRP1), a low specificity receptor, has been isolated [129,130]. LRP1 can bind to multiple targets. It is a type I transmembrane receptor of 600 kDa, composed of five subunits [130], which is abundantly expressed in hepatocytes, neurons, smooth muscle cells, fibroblasts, and cholangiocytes [119,124]. In hepatocytes, it is involved in the uptake of lipoproteins containing triglycerides and cholesterol through an endocytic pathway. LRP1 has also been shown to take part in several cellular processes, including cell migration, survival, motility, and differentiation, and it has been proven to play a role in different pathologies such as thrombosis, fibrinolysis, and atherosclerosis [131]. Other than binding hLf, this receptor has a diverse array of functions: lipoprotein metabolism, proteinase metabolism, activation of lysosomal enzymes, cellular entry for viruses and toxins [129]. Regarding the interaction between the viral particle and LRP1, this receptor is emerging as a new receptor for different viruses. In fact, different species of human rhinoviruses use LRP1 to enter inside the cells [132,133]. LRP1 was also found to be bound to the HIV-1 transactivator (Tat) protein. The binding of Tat protein to this receptor promotes the efficient uptake of Tat by neurons. The neuronal uptake of Tat, through LRP-mediated endocytosis, inhibits neuronal clearance of the physiological ligands of LRP, such as amyloid precursor protein and amyloid β-protein, contributing to neurological disorders associated to HIV-1 [134]. Another virus that uses LRP1 to enter the cells is HCV. Experimental and clinical data suggest that LRP1 is a co-receptor for HCV. In fact, HCV RNA levels in primary hepatocytes are correlated to LRP1 mRNA expression [135]. Liver is specialized to produce and secrete lipoproteins for transporting various lipid species, such as triglycerides that from the lipid droplets (LDs) are hydrolyzed into fatty acids and re-esterified within the endoplasmic reticulum (ER) in triacylglycerides (TAGs) that are packaged into very-low-density lipoproteins (VLDLs). LDs are also a stock of membrane lipid precursors and of signaling lipids [136]. These kinds of functions make LDs an attractive target for pathogens to support their replication. For that reason, several viruses, including HCV, exploit this organelle to complete different steps of their proliferation [137]. HCV uses a combination of entry factors including lipid and lipoprotein receptors that is quite unique to the hepatocyte, for example LRP1 [138]. In fact, HCV exists as a lipoprotein-virus hybrid lipoviroparticle (LVP). The secretion of mature VLDL particles occurs through the Golgi secretory pathway. HCV virions latch onto or fuse with a nascent VLDL particle in either the ER or the Golgi compartment, resulting in the generation of LVPs. HCV LVPs assemble in the ER and are transported to the Golgi compartment in COPII vesicles to enter the Golgi secretory route [139]. After partial hydrolysis of the triglycerides present in VLDL, HCV associated with VLDL binds target cells through LPR1, thus infecting hepatocytes [140]. Concerning other hLfRs, CD14 has been exclusively found on monocytes, asialoglycoprotein receptor (ASGPR) in the liver, and nucleolin in lymphocytes [119,141]. Interestingly, in addition to ITLN1, the surface nucleolin is involved in the ability of hLf to enter the nucleus [115,116,142]. Therefore, due to receptor specificity, hLf can exert several different functions depending on the cell system it acts upon. Regarding bLf, its interaction with hLfRs was first described by Shin et al. [143], and its nuclear localization in human enterocytes was then observed [144], thus strengthening and partially explaining how bLf could act as a potent bioequivalent of the human homologue.

## 5. Antiviral Activity of Lactoferrin in Apo- and Metal-Saturated Forms

The antiviral activity of hLf was first demonstrated in mice infected with the polycythemia-inducing strain of the Friend virus complex (FVC-P) [145]. An antiviral activity of both hLf and bLf against enveloped and naked viruses has been shown [109,146]. In some in vitro studies, bLf in apo- and in metal-saturated forms exerts a similar antiviral activity thus indicating that Lf does not only inhibit viral infection through its iron chelating property but also hinders viral attachment to the target host cells through its competitive binding [109,147,148,149,150,151]. In particular, Lf can interact with negatively charged compounds such as glycosaminoglycans (GAGs), thus inhibiting virus-receptor interaction [152]. In addition to interacting with GAGs, Lf prevents viral infections by binding to dendritic cell-specific intercellular adhesion molecule 3-grabbing non-integrin (DC-SIGN) and LDL receptors [153,154]. Moreover, Lf exerts an antiviral activity against cytomegalovirus (CMV), herpes simplex virus (HSV), HIV, rotavirus, poliovirus (PV), respiratory syncytial virus (RSV), HBV, HCV, parainfluenza virus (PIV), alphavirus, hantavirus, human papillomavirus (HPV), feline calicivirus (FCV), adenovirus, enterovirus 71 (EV71), echovirus 6, influenza A virus, Japanese encephalitis virus, and tomato yellow leaf curl virus (TYLCV) [109,146]. The authors of these two reviews agree that the antiviral effect of Lf occurs in the early phase of infection, preventing the entry of viral particles into the host cells, either by blocking cellular receptors or by directly binding to the viral particles. The capability of Lf to exert antiviral activity, by binding to host cells or viral particles or both, strengthens the idea that this glycoprotein is “an important brick in the mucosal wall, effective against viral attacks” [103].

## 6. Iron Proteins and Lactoferrin in Viral Hepatitis

As previously reported, in healthy conditions, most of the body iron is present in a protein-bound form, such as Tf- or Lf-bound iron, to maintain it in an unreactive form, whilst, in pathological condition, i.e., infection and/or inflammation, reactive free iron can act as a catalyst for the production of ROS. Iron is also crucial in many aspects of the innate immune response, where pathogen and host establish a battle for its bioavailability. The host seeks to reduce the amount of free harmful iron available for pathogens by sequestering it inside the cell, mainly in protein-bound form, in adequate quantities for normal immune function. On the other hand, iron overload disorders lead to iron oversaturation of Tf and Lf as well as to an increased susceptibility not only to bacterial [155] but also to viral infections [70]. As matter of fact, viral replication is linked to an increased cellular metabolism, necessary to copy viral genomes and to synthesize viral proteins. Infection processes require iron, so the host must be replete in iron to allow the efficient propagation of a virus [82]. Chronic iron deposition in the liver induces hemochromatosis correlated with tissue damage, cirrhosis, and HCC. Moreover, ROS, produced by the excess of iron, can generate an inflammatory process that stops the physiological hepatic function [156]. Severe liver damage can be provoked by hepatic viral infections generally sustained by iron. From this point of view, an iron-binding protein as Lf could represent an efficient treatment in inhibiting viral infection. Indeed, Lf through its anti-inflammatory activity can indirectly modulate the proteins involved in iron homeostasis including Fpn [44] and hepcidin [112]. Moreover, Lf could also directly increase the expression of Fpn which, in turn, decreases intracellular iron overload thus depriving viral particles and host cells from this essential element [44]. On the other hand, Lf exerts its antiviral activity not only by regulating iron homeostasis proteins, but also by interacting with viral molecules and/or cellular GAG [109,146]. Concerning the efficacy of Lf against HBV, the pre-incubation of the cells with bLf or hLf was required to prevent HBV infection, while the pre-incubation of HBV with bLf or hLf had no inhibitory effect on the infection rate, thus indicating that the sole interaction of Lf with the cell surface is responsible for an antiviral effect [157]. Concerning the iron proteins, they are modulated according to the virus type and the stage of infection. Regarding HBV, during the acute viremic phase, no significant variation in hepcidin expression as well as in serum Ftn and iron levels was reported [81]. Conversely, two studies on the expression of iron-handling proteins in chronic HBV-infected patients with or without cirrhosis as well as with cirrhosis and HCC were carried out [41,158]. Wang et al. [158], showed that serum hepcidin levels were slightly, yet significantly, increased in patients presenting no cirrhotic process and in those with HCC, but not in those with cirrhosis, when compared to healthy controls. Surprisingly, the increase of hepcidin levels was not correlated with a decrease of serum iron and Ftn, which, on the contrary, significantly increased in all groups compared to healthy subjects [158]. In the study by Gao and colleagues [41], serum hepcidin levels were decreased in relation with an increase of serum Ftn in all three groups, whereas a significant increase of serum iron content was observed only in patients without cirrhosis [41]. Regarding Tf, despite a significant increase in its iron saturation rate for all three groups when compared to the controls, no significant difference in its expression was recorded [41]. Altogether, these two studies reported a similar trend of iron proteins in chronic HBV patients, with a global increase in serum iron and Ftn content. Of note, the conflicting data on hepcidin levels, between the two studies, were related to the control groups’ values rather than to differences in the HBV groups. In fact, HBV groups present similar serum hepcidin levels in their absolute values [41,158]. Differently from HBV and other viruses positively utilizing intracellular iron [41], HCV is damaged by intracellular iron overload [70,159]. During the first steps of infection, HCV enters in hepatocytes through an orchestrated process starting from the binding of the viral particles to receptors with co-receptors. The initial capture of HCV particles by GAGs and/or lipoprotein receptors is followed by coordinated interactions with the CD81 tetraspanin, the tight junction proteins Claudin-1 and Occludin [160]. The process of viral entry into cells can be divided into a three-step process. Firstly, HCV recognizes a target cell by binding to the mannose-binding lectins L-SIGN, mainly expressed on the endothelium of liver and DC-SIGN, mainly present on dendritic cells. Both proteins are believed to act as HCV capture receptors. Then, the viral glycoproteins interact with the CD81 tetraspanin and lipoprotein receptors, moving the virus from the surface to side gradually. In the end, tight junction proteins may be used to sustain HCV entry by inducing clathrin-mediated endocytosis. This tight network of receptor interactions leads to uptake and cellular internalization of HCV through a process of clathrin-dependent endocytosis [161]. Besides, the combination of both hepatic iron accumulation and HCV proteins leads to the production of large quantities of the toxic hydroxyl radical (·OH). ·OH induces hepatic injury and, through its reaction with guanine, forms mutagenic bases including 8-hydroxy-2-deoxyguanosine (8-oxodG) [162]. The expression levels of 8-oxodG in liver tissue were examined in HCV patients as a marker of oxidative DNA damage and were found to be elevated approximately 10-fold compared to non-HCV infected control patients [163].

**Figure 3:**
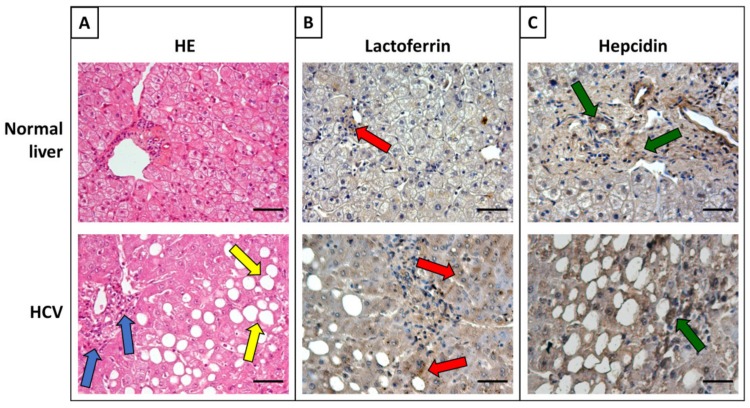
Morphological evaluation and immunohistochemistry of lactoferrin and hepcidin in normal and HCV-infected liver performed according to the procedures reported in our previous papers [124,176,177]. (**A**) Hematoxylin and eosin stain (HE): morphology of a normal liver, with a regular histological appearance and a liver with chronic hepatitis C infection (HCV), with portal and periportal fibrosis and inflammation (blue arrows) together with steatosis (yellow arrows). (**B**) Immunohistochemistry: HCV infection induces a significant up-regulation of lactoferrin synthesis (red arrows), probably related to the activation of the inflammatory process. (**C**) Immunohistochemistry: HCV infection significantly down-regulates the expression of hepcidin compared to normal liver (green arrows). OM 20×. Scale bar = 50 μm.

In the first phase of infection, hepcidin is up-expressed and HCV uses TfR1 as a cargo for its internalization in endosomal compartments [91]. In the chronic phase, HCV tries to counteract cellular defenses through a non-canonical mechanism of iron homeostasis consisting in the down-regulation of hepcidin [91] (Figure 3). Hepcidin inhibition causes an up-regulation of Fpn with consequent increase of iron export. The down-regulation of hepcidin as well as TfR1 and DMT1 hinders iron import into host cells causing HCV progression and predisposition to liver fibrosis, cirrhosis and HCC [91,164] (Figure 3). Notably, Lf expression results up-regulated in HCV-infected tissues, in agreement with the high infiltration rate of neutrophils (Figure 3) [165].

In in vitro experiments, differently from the inhibition of HBV entry, which occurs only through the competitive binding of Lf with cellular GAGs [166], Lf inhibits HCV also through its direct interaction with viral molecules [167,168]. In particular, the binding of Lf with GAGs, and more specifically with heparan sulfate (HS), prevents the first contact between HCV and host cells, thus inhibiting infection [109]. Lf is also able to bind to the HCV envelope proteins E1 and E2 [167], thus inhibiting the interaction between the viral particles and cellular receptors. Moreover, further in vitro investigations revealed a HCV intracellular target for hLf, the ATPase/Helicase non-structural 3 (NS3) protein [169]. This interaction is mediated by a direct and specific binding between hLf and an allosteric site on NS3, thus indicating an additional antiviral mechanism of action by which hLf inhibits the intracellular HCV replication [169]. Other than these in vitro results, the effect of Lf administration in patients with chronic HCV has been reported in six clinical trials [170,171,172,173,174,175]. The first pilot study on eleven patients with chronic hepatitis C receiving for 8-weeks 1.8 or 3.6 g/day bLf showed a decrease in serum alanine transaminase (ALT) and HCV RNA levels, suggesting that bLf could be efficient against HCV infections [170]. Successively, another trial by the same group was designed to assess the relationship between the dose and the effect of bLf on serum ALT and HCV RNA levels in forty-five patients with chronic hepatitis C who orally received 1.8, 3.6, and 7.2 g/day bLf for 8-weeks [171]. However, no correlation between the dose and the effect of bLf was recorded among the three different groups [171]. Although the quantity of bLf administered in this clinical trial was high, an excellent tolerance for bLf was observed [171]. In 2006, conflicting results were reported by Ueno et al. [172] who demonstrated that oral administration of 1.8 g/day of bLf for 12 weeks on ninety-seven patients did not show any significant efficacy in reducing ALT and in serum HCV RNA levels with respect to one hundred one treated with placebo. As the treatments of HCV are costly, long-life, and have adverse effects, a combination therapy of bLf with interferon [173,174] or with interferon plus ribavirin [175] has been tested to ameliorate the therapeutic strategy. From these studies, bLf seemed to contribute to the efficacy of the combined therapy by decreasing HCV RNA titer. 

## 7. Conclusions

The liver contains the largest resident reticulo-endothelial cell network in the body, which composes the antigenic environment of the liver. This environment ensures that if a pathogen enters the liver, an acute immune response ensues, leading to self-limiting inflammation and to repair of the tissue. In instances in which inflammation is chronic, substantial cellular recruitment and tissue damage may result. In the presence of sustained inflammation, this damage is not always repaired properly, resulting in fibrosis and dysfunction. But also, a failure to initiate an appropriate immune response in the presence of viral infection or cancer can result in the development of chronic disease and organ failure. In particular, hepatitis represents a complex disease, one of the major public health problems worldwide. For example, as much as 40% of men with perinatally acquired hepatitis B virus infection will die of liver cirrhosis or hepatocellular carcinoma. As described for other inflammatory diseases, including Crohn’s disease and ulcerative colitis, hepatitis is associated to the activation and maintenance of inflammatory processes, which became one of the principal contributory causes to organ failure and development of concurrent pathologies, including cancer. Moreover, as emerging for this review, iron dysregulation induced by, but also inductor of, inflammatory disorders plays a crucial role in the activation and progression of hepatic viral infection. Indeed, HBV and HCV, even if through opposite paths, take control of iron-handling proteins, thus regulating intracellular iron content for their own needs. Altogether, viral infection, iron overload, and chronic inflammatory processes have been associated to increased morbidity and mortality of hepatitis.

For that reason, antiviral treatments able to exert multi-targeting activities against viral infection and replication as well as against inflammatory and iron dysregulation represent a promising tool to counteract hepatic failure and injury. In this respect, a protein belonging to the innate immunity, Lf, presents a well-known intrinsic antiviral activity, which makes this protein an interesting candidate for application as a drug carrier in hepatitis. Overall, the capacity of Lf to act as an anti-inflammatory agent is based on its ability to down-regulate pro-inflammatory cytokines involved in the complex orchestration of iron homeostasis, mediated by the major iron-handling proteins, such as TfR1, Ftn, Fpn, and Heph/Cp. Of note, Lf in physiological conditions does not exert any effect on host cells, whereas in pathological ones possesses “the ability to sense the immune activation status of an organism and act accordingly” [178].

Altogether, different studies are contributing to understand the fine-tuning of immune responses in the liver from homeostasis to disease, starting to consider that some of these aspects will represent novel targets for future therapies in acute and chronic liver diseases.
